# Experimental Infection of North American Sheep with *Ehrlichia ruminantium*

**DOI:** 10.3390/pathogens10040451

**Published:** 2021-04-09

**Authors:** Arathy Nair, Paidashe Hove, Huitao Liu, Ying Wang, Ada G. Cino-Ozuna, Jamie Henningson, Charan K. Ganta, Roman R. Ganta

**Affiliations:** 1 Center of Excellence for Vector-Borne Diseases (CEVBD), Department of Diagnostic Medicine/Pathobiology, Kansas State University, Manhattan, KS 66506, USA; arathy@vet.k-state.edu (A.N.); phove@vet.k-state.edu (P.H.); hliu@vet.k-state.edu (H.L.); yivawang@gmail.com (Y.W.); 2 Department of Pathobiology, School of Veterinary Medicine, St. George’s University, Grenada, West Indies; 3 Kansas State Veterinary Diagnostic Laboratory, College of Veterinary Medicine, Kansas State University, Manhattan, KS 66506, USA; acinooz@okstate.edu (A.G.C.-O.); heningsn@vet.k-state.edu (J.H.); ckganta@ksu.edu (C.K.G.)

**Keywords:** rickettsials, *Anaplasmataceae* pathogens, tick-borne diseases, heartwater, *Cowdria ruminantium*

## Abstract

*Ehrlichia ruminantium*, a tick-borne rickettsial, causes heartwater in ruminants resulting from vascular damage. Severity of heartwater varies greatly in ruminant species and breeds, age of animals and for diverse geographic *E*. *ruminantium* strains. *E. ruminantium* and a tick vector, *Amblyomma variegatum*, originating from Africa, are well established in certain Caribbean islands two centuries ago. Besides the possibility of introduction of heartwater through African exotic animal importation, presence of the pathogen, and the tick vector in the Caribbean pose a high risk to ruminants in the USA and other western hemisphere countries. Scientific evidence supporting the heartwater threat to nonendemic regions, however, is lacking. We describe the first infection study in sheep reared in the USA with seven *E. ruminantium* strains. All infected sheep exhibited clinical signs characteristic of subacute to subclinical disease, which included labored breathing, depression, coughing, and nasal discharges. Gross and microscopic lesions consistent with heartwater disease including edema and hemorrhage were observed in several organs. Pathogen-specific IgG antibody response was detected in animals infected with all seven strains, while molecular analysis confirmed the pathogen presence only when infected with in vitro cultures. This is the first infection study demonstrating severe heartwater in sheep reared in North America.

## 1. Introduction

Heartwater is a high economic impact tick-borne disease affecting a wide range of ruminants including cattle, sheep, goats, several species of antelope and wild ruminants. The disease is caused by the Gram negative rickettsial pathogen, *Ehrlichia ruminantium* [1,2]. Characteristic symptoms of heartwater may include fever, respiratory distress, edema, and fluid accumulation in lungs, heart, thorax, peritoneum and liver. Edema and hemorrhages are also frequently noted in various tissues during histopathological examinations [3,4]. Clinical outcomes of heartwater vary in animal species and are also based on the age and breed of animals and their geographic origins [5]. Further, *E. ruminantium* strains differ in causing variable levels of pathogenesis [4]. Animals with a rare peracute form of the disease exhibit sudden death following high fever or animals may be found dead without any clinical signs, while animals with the acute form may present a few days of high fever, depression, cough, heavy breathing, and neurological signs, which may result in high mortality [6,7]. Subacute and subclinical forms of the disease cause moderate to high morbidity with clinical signs ranging from a few days of fever, labored breathing and cough followed by recovery or death. Subclinical heartwater disease is characterized by transient fever and is reported to be less severe in some breeds of sheep, cattle or antelope with natural resistance to the disease [7]. *Ehrlichia ruminantium* multiplies primarily in vascular endothelium and causes vascular damage leading to edema and hemorrhages in various organs of infected animals [8].

The disease is responsible for a high economic burden, particularly in the disease-endemic regions of the world, while also posing a potential risk to naïve ruminant populations if introduced into regions currently nonendemic for the disease. *Ehrlichia ruminantium* is mainly endemic in sub-Saharan Africa and some islands in the Indian Ocean [5,9]. It is transmitted by *Amblyomma* species ticks, primarily *A. variegatum* and *A. hebraeum* [3,10]. *Amblyomma variegatum* and *E. ruminantium* are thought to have been introduced from northern part of Africa to the French Caribbean islands (Guadeloupe and Marie-Galante) and spread to another nearby island (Antigua) about two hundred years ago [11,12,13], which is supported by the molecular evidence [14]. Notably, despite the spread of *A. variegatum* to several Caribbean islands, which is documented about one and a half centuries after the first introduction, the disease is restricted only to three nearby islands and with no documented major disease outbreaks [11]. *Ehrlichia ruminantium* strains in the Caribbean are postulated to be avirulent and over the years have become nonpathogenic [15]. It is also hypothesized that ruminant populations in the Caribbean are innately resistant to the disease [13,16]. Consistent with this hypothesis, recent molecular studies suggest that Panola Mountain *Ehrlichia*, a suspected new strain of *E. ruminantium*, is found to be prevalent in *Amblyomma maculatum* from the USA and *A. variegatum* from the Caribbean and Africa, while causing minimal disease in ruminant populations [17,18]. Little published evidence supports the risk of spreading *E. ruminantium* beyond the three Caribbean islands; however, its presence along with the wide-spread distribution of *A. variegatum* in several Caribbean islands is perceived as a constant threat to domestic and wild ruminant populations in the Americas [19,20]. Further, *A. maculatum* from the USA is identified as a competent vector for the pathogen [21]. 

The United States Department of Agriculture (USDA) classified heartwater and *A. variegatum* on the list of high consequence and high-risk foreign animal diseases and pests (https://www.hsdl.org/?view&did=735649; accessed on 8 April 2021). Further, the USDA recently conducted a detailed scientific analysis workshop to provide a foundation for controlling heartwater (https://www.star-idaz.net/app/uploads/2019/11/Heartwater-Gap-Analysis-Report-2018-Final.pdf; accessed on 8 April 2021). Multidisciplinary scientists from several continents, including those from Africa, Europe, South and North America, discussed current scientific knowledge of the disease to identify its potential threats to livestock and to define research needs and priorities. The scientific team also discussed the available countermeasures to contain and mitigate threats posed by the disease. The analysis identified knowledge gaps required in advancing the development of control strategies in order to prevent heartwater outbreaks. Research priorities proposed by the scientific team included the understanding of (1) bacterial infection and pathogenesis in animals, (2) transmission and epidemiology, and (3) developing improved countermeasures such as diagnostics, vaccines, and tick control to yield significant improvements in mitigating heartwater outbreaks.

Our current study was focused on addressing the first major goal of the above identified research priorities. In particular, with a recent USDA regulatory change permitting heartwater research with live organisms on the mainland USA, we could carry out the first pathogenesis study in sheep in an effort to gain the first evidence in determining the disease risks to ruminant populations in the USA. Specifically, we describe *E. ruminantium* infection in two different breeds of sheep reared in mainland USA and assessed pathogenesis using seven different strains of the pathogen. We observed that all seven strains of *E. ruminantium* caused disease with a high degree of severity in sheep.

## 2. Results

### 2.1. E. ruminantium Infection with Blood Stabilates

#### 2.1.1. Blood Stabilate Infections Induced Clinical Disease Consistent with Heartwater in North American-Raised White Dorper Sheep

In an effort to define how heartwater impacts ruminants in North America, we used six-month old White Dorper breed male sheep for infection (Figure S1). Blood stabilates representing six different strains of *E. ruminantium* were used to infect six sheep, as all strains are known to produce severe heartwater in ruminants in endemic regions of sub-Saharan Africa [21]. All animals prior to infection challenge tested negative for exposure to *Ehrlichia* species as judged by genus-specific PCR and by ELISA analysis (not shown). Following infection challenge, the animals were monitored daily for clinical response for up to 28 days (Table 1). Clinical signs characteristic for heartwater were more evident after two weeks following the stabilate inoculations in all sheep and the clinical signs persisted for several days, while fever was observed only for one to two days. The most noticeable clinical signs observed in all animals were labored breathing, depression, persistent cough, and mucopurulent discharges. The respiratory rate in animals increased to 50–65 breaths per min compared to the normal range of 16–34 breaths per min [22].

#### 2.1.2. Gross Pathological Lesions Consistent with Heartwater Were Observed in Sheep Infected with *E. ruminantium* Blood Stabilates

**Figure 1:**
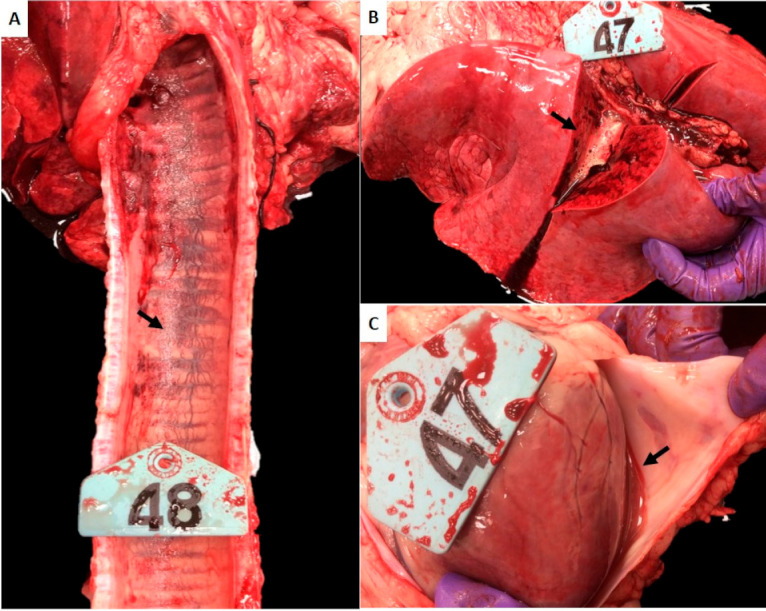
Gross pathological lesions observed in White Dorper breed sheep. Tissue samples collected on day 28 post infection (challenge) were assessed in sheep infected with *E. ruminantium* blood stabilates. (**A**) Trachea, submucosal congestion and hemorrhage (arrow). (**B**) Lung, diffuse and severe congestion and edema. Note the copious amounts of foamy fluid oozing from the cut section (arrow). (**C**) Heart, pericardial serosanguineous fluid (arrow).

Gross lesions were observed in all animals (Table 2 and Figure 1). Consistent with the clinical respiratory abnormalities noted, the major lesions at necropsy in all animals included multifocal mild to moderate hemorrage in the submucosa of the larynx and trachea (Figure 1A), and severe, diffuse congestion and edema in the lungs (Figure 1B). Hemorrhage in the larynx and trachea was more prominent in the Malelane strain-infected sheep (Figure 1A). Serosanguineous pericardial transudate was observed in animals infected with the Pokuase and Nigeria strains (Figure 1C) where the clinical signs persisted for the longest duration (8 days and 13 days, respectively). The Highway strain-infected animal was less severely affected exhibiting only two days of respiratory distress and gross lesions present in this animal included pulmonary congestion and edema and included enlarged, congested, and edematous mediastinal lymph node. The Crystal Springs strain-infected animal had edematous tracheobronchial lymph nodes.

#### 2.1.3. Histopathology Reveals Lesions Typically Observed in Heartwater in Sheep Infected with *E. ruminantium* Blood Stabilates

**Figure 2:**
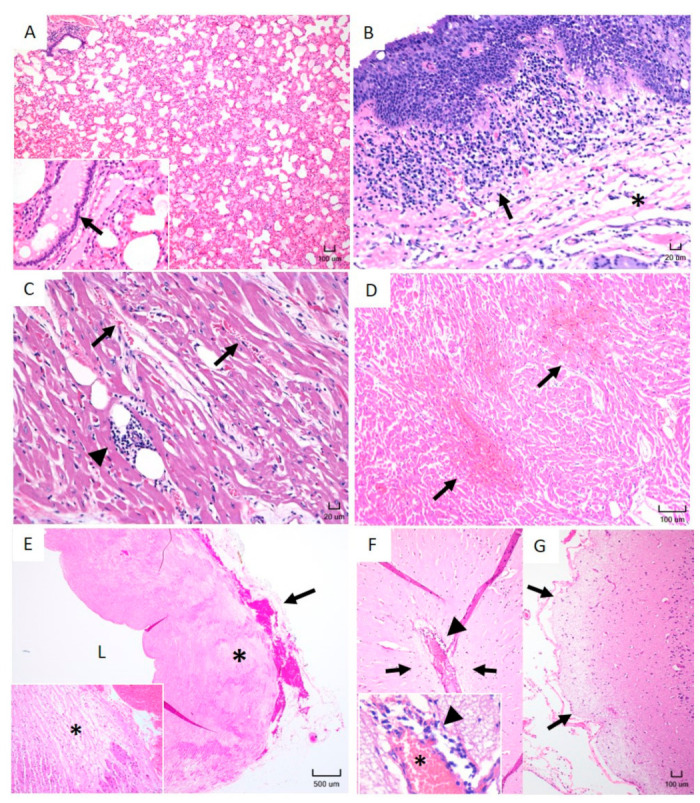
Histopathological lesions observed in White Dorper breed sheep at 28 days following *E. ruminantium* blood stabilate infection. (**A**) Lung, moderate interstitial congestion and edema. 10× magnification, H&E. Insert: Higher magnification of a bronchiole filled with edema fluid. (**B**) Trachea, severe lymphoplasmacytic tracheitis (arrow) with severe edema (asterisk). 20× magnification, H&E. (**C**) Heart, mild multifocal lymphoplasmacytic myocarditis (arrowhead) with mild multifocal hemorrhage (black arrows). 20× magnification, H&E. (**D**) Heart, severe myocardial edema and hemorrhage (arrows). 10× magnification, H&E. (**E**) Aorta, tunica media edema (asterisk) and adventitial hemorrhage (arrow). The lumen of the vessel is marked with the letter “L”. 2× magnification, H&E. Insert: Higher magnification of the blood vessel wall edema (asterisk). (**F**,**G**) The two panels depict mild (**F**) to moderate (**G**) cortical cerebral edema (arrows). 10× magnification, H&E. Insert: Higher magnification of a cerebral meningeal vessel (asterisk) with lymphoplasmacytic perivascular infiltrates (arrowhead).

Histopathological lesions with considerable cellular infiltrations, edema, and hemorrhage were observed in various tissues, including the lungs, trachea, heart, aorta, and brain (Figure 2). Lungs of all infected animals exhibited interstitial congestion and edema with multifocal moderate mononuclear perivascular cellular infiltration (Figure 2A). Submucosal tracheitis with moderate lymphoplasmacytic infiltration, mild epithelial hyperplasia, and severe edema were also observed in all infected animals (Figure 2B). Mild to moderate multifocal lymphoplasmacytic infiltration was evident in the myocardium of the animals along with multifocal hemorrhage (Figure 2C,D). Moderate edema and periaortic hemorrhage were observed in all infected animals (Figure 2E). Moderate (Figure 2F) to mild (Figure 2G) cortical cerebral edema with lymphocytic meningitis and mononuclear cellular infiltration were noted in animals infected with Crystal Springs, Highway, and Malelane strains.

#### 2.1.4. Blood Stabilate-Infected Sheep Induced a Bacterial Antigen-Specific IgG Response While Testing Negative for DNA

**Figure 3:**
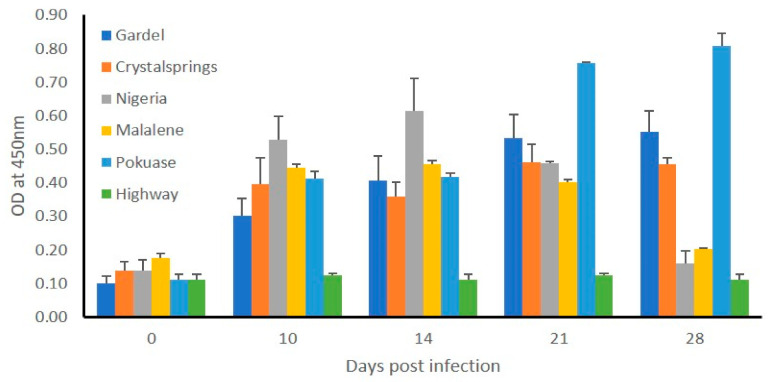
*E. ruminantium*-specific IgG response in sheep infected with blood stabilates. Antigen-specific IgG antibodies were measured in the plasma samples collected at day zero (prior to infection) and multiple time points post infection challenge by ELISA analysis. Average absorbance values of plasma collected from each sheep at each time point assessed in triplicate wells were plotted against the blood sampling days. Whole cell lysate prepared from culture-derived purified *E. ruminantium* Crystal Springs strain was used to coat the plates.

Total DNA recovered from sheep blood collected from the animals twice a week and several tissue samples collected during necropsy on day 28 were assessed for the presence of *E. ruminantium* DNA by nested PCR. Despite the clinical signs and gross and histopathological observations consistent with heartwater, all samples tested negative for the pathogen-specific DNA and by culture recovery. The presence of IgG antibodies was assessed by ELISA in the plasma collected from all sheep using total bacterial antigens prepared from in vitro cultured Crystal Springs strain (Figure 3). All infected sheep responded to infection by inducing the synthesis of *E. ruminantium*-specific IgG antibody with the exception of the animal infected with the Highway strain blood stabilate. The IgG levels were significantly higher in the plasma samples assessed starting from day seven post infection compared to day zero. The antibody levels increased or remained high until day 21 and declined for sheep infected with Nigeria and Malelane strains blood stabilates.

### 2.2. Infection Assessed in Sheep with In Vitro Cultured E. ruminantium Strains

#### 2.2.1. Clinical Disease in Sheep Infected with In Vitro Cultured *E. ruminantium* Strains

To determine if disease severity differs with the type of infection inoculum and if sheep breed and sex also contribute to the disease outcome, we repeated the infection study using three in vitro cultured virulent strains of *E. ruminantium*; Mbizi, Crystal Springs, and Highway (Figure S2) and using six-month-old Katahdin and Romanov cross-bred female sheep (Figure S3). Clinical response in this group of sheep is similar to that observed with blood stabilate inocula. The signs included occasional fever, labored breathing, cough, and depression (Table 3). The clinical signs were independent of the strain of *E. ruminantium* used. One notable difference was that the clinical signs appeared almost a week earlier compared to infections assessed using blood stabilate inocula.

#### 2.2.2. Gross Lesions in Several Tissues Were Evident in Sheep Infected with In Vitro Cultured *E. ruminantium*

**Figure 4:**
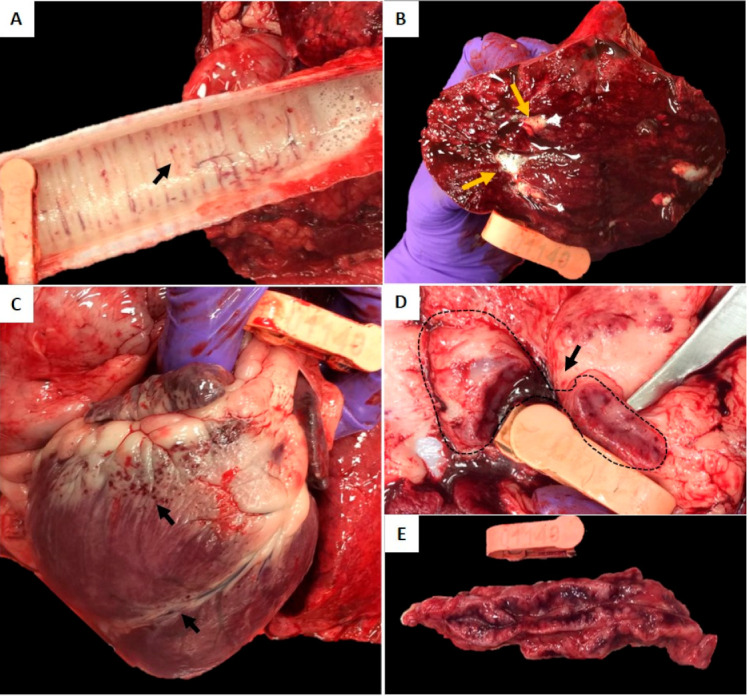
Gross pathological lesions observed in sheep infected with in vitro cultured *E. ruminantium* strains. (**A**) Trachea: Mild petechial submucosal hemorrhage (arrow). (**B**) Lung: Severe and diffuse congestion and edema. Note the foamy serosanguineous fluid oozing from the cut section of lung (orange arrows). (**C**) Heart: Subepicardial petechial hemorrhage (arrows). (**D**) Cross section of a congested and hemorrhagic supramammary lymph node (dotted line and arrow). (**E**) Enlarged and hemorrhagic mediastinal lymph node.

Gross pathology observations on day 28 for the in vitro cultured *E. ruminantium* inoculations were also consistent for heartwater disease (Table 4 and Figure 4). As with the prior infection study, the most severe lesions in all sheep included submucosal congestion and hemorrhage in trachea and larynx, and congestion and edema in lungs (Figure 4A,B). Similarly, hemorrhage and edema were evident in the pericardial surface of the heart, mammary glands, and lymph nodes in an Mbizi infected animal. Enlargement, congestion, and edema of mediastinal and aortic lymph nodes were observed in all animals. (Typical gross lesions for heart and lymph nodes are shown in Figure 4C–E).

#### 2.2.3. Histopathology Supported the Presence of Extensive Lesions Consistent with Heartwater in Sheep Infected with In Vitro Cultured *E. ruminantium* Strains

**Figure 5:**
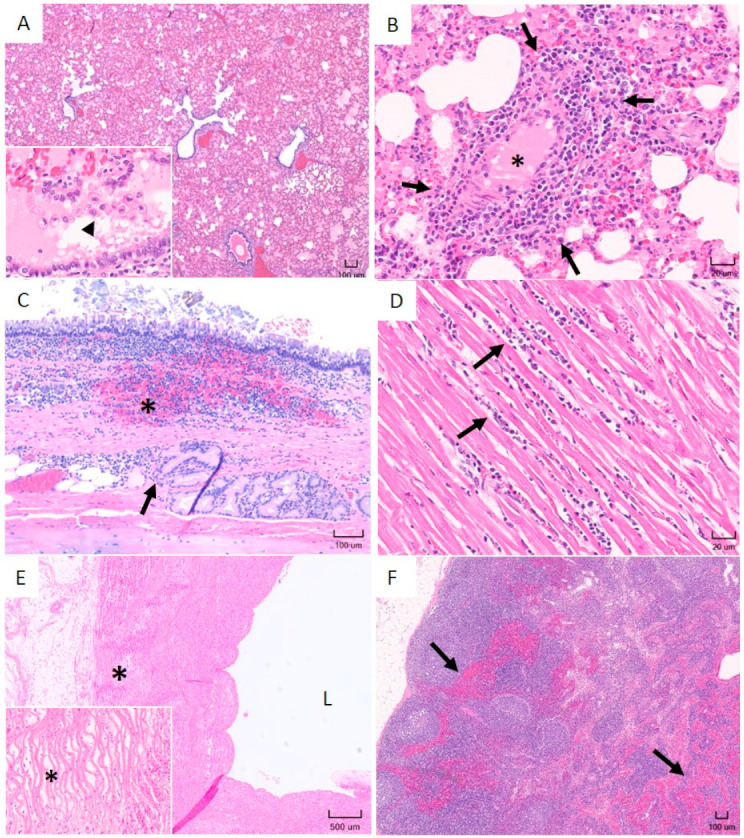
Histopathological lesions observed in sheep infected with in vitro cultured *E. ruminantium* strains. (**A**) Lung: Moderate interstitial congestion and edema. 4× magnification, H&E. Insert: Higher magnification of a bronchiole with edema fluid and several foamy macrophages (arrowhead). (**B**) Lung: Perivascular (arrows) to interstitial lymphoplasmacytic pneumonia (lumen of blood vessel marked with asterisk). 40× magnification, H&E. (**C**) Trachea: Submucosal hemorrhage (asterisk) with lymphoplasmacytic tracheitis. Arrow points to inflammation extending deeper in the submucosa, around submucosal glands. 10× magnification, H&E. (**D**) Heart: Moderate multifocal lymphoplasmacytic myocarditis (arrows). 40× magnification, H&E. (**E**) Aorta: Moderate vascular wall edema. “L” denotes the lumen of the vessel. 2× magnification, H&E. Insert: Higher magnification of vascular wall edema. Note separation of elastic fibers by edema fluid (asterisk). (**F**) Lymph node: Extensive interstitial congestion and hemorrhage (arrows). 4× magnification, H&E.

Extensive hemorrhage, edema, and cellular infiltrations were present in multiple tissues (Figure 5). The most consistent microscopic lesions were detected in the lung, trachea, heart, aorta and lymph nodes. Pulmonary congestion, hemorrhage, and edema with perivascular to interstitial infiltration of mononuclear cells were seen in all animals (Figure 5A,B). Submucosal hemorrhage and mild lymphoplasmacytic cellular infiltrates were detected in tracheae of infected sheep (Figure 5C). Lymphoplasmacytic myocarditis and multifocal myocardial hemorrhage were detected in the heart (Figure 5D). Mild tunica media edema was observed in the aorta (Figure 5E). Extensive edema and hemorrhage were evident in the enlarged mediastinal, aortic, and mammary lymph nodes (Figure 5F).

#### 2.2.4. Infected Animals Developed IgG Response to *E. ruminantium* Infection, and Several Tissue Samples Tested Positive for Bacterial DNA

**Figure 6:**
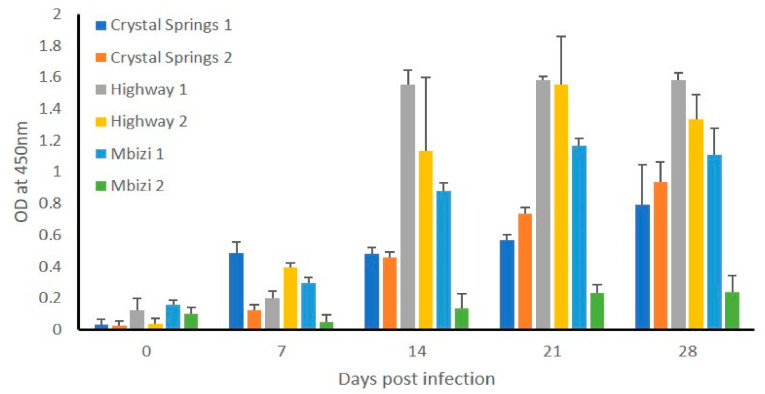
*E. ruminantium*-specific IgG response in sheep infected with in vitro cultured *E. ruminantium* strains. Antigen-specific IgG antibodies were measured in the plasma samples collected at multiple time points post infection challenge by ELISA analysis. Average absorbance values of plasma collected from each sheep at each time point assessed in triplicate wells were plotted against the blood sampling days. The day zero represents plasma samples collected from sheep prior to infection challenges. *E. ruminantium* Crystal Springs strain culture-derived purified antigens were coated on the ELISA plates.

Five of the six animals responded by producing *E. ruminantium*-specific IgG antibodies starting from day seven post infection, as judged by ELISA analysis (Figure 6). The antibody levels steadily increased and were significantly higher starting from day 14, relative to day zero. Total DNA recovered from blood sampled at different times and from various tissues collected at the time were assessed by the nested PCR assay for the presence of *E. ruminantium* DNA. All six animals tested positive for the bacterial DNA in tissue samples of recovered; lungs, heart, aorta, and mediastinal and aortic lymph nodes (Table 5), while blood samples tested negative.

## 3. Discussion

Pathogenesis varies for different *E. ruminantium* strains circulating in Africa and in the Caribbean. To gain insights into heartwater pathogenesis in ruminants reared in a nonendemic region; the USA mainland, our current study focused on performing the first ever infection study in 6-month-old sheep and we used two breeds of animals representing both sexes. We observed clinical and pathological changes characteristic of heartwater disease in both White Dorper breed males and similarly in the cross bred Katahdin and Romanov female sheep while not resulting in fatalities. All animals exhibited clinical signs as well as gross and histopathological lesions consistent with heartwater. While *E. ruminantium* infection induced an IgG antibody response in most animals, the pathogen was cleared to undetectable levels in White Dorper breed, but not in of Katahdin and Romanov crossbreed sheep when assessed by a molecular method. We attributed this difference to the type of inoculum used. In particular, infection cleared from White Dorper sheep to undetectable levels when blood stabilates were used as the infection source, while the pathogen persisted in various tissues of the infected animals when in vitro cultured organisms were used as infection inocula. While one animal per strain was used in the first experiment using six different pathogen strains, clinical signs as well as gross and histopathological lesions remained very similar in all animals. The clinical and pathology data are also similar to the second infection experiments where we used three strains of freshly cultured *E. ruminantium*. In the second experiment, we also repeated infection with two of the three strains assessed in the first experiment. Independent of the seven different strains used and also independent of the inocula used, all animals exhibited very similar pathogenesis, as evidenced by the clinical and pathological observations consistent for the subacute and subclinical forms of the disease. 

We monitored animals for only 28 days in the current study during which time all sheep exhibited clinical signs by the second week or slightly thereafter, but all animals survived during the four-week assessment time. It is possible that we may have noted more robust clinical signs such as fatalities if the study period was extended. Also, likely that if different age group animals are used, the disease outcomes may differ. Indeed, a study carried out in Namibia demonstrated that Dorper sheep develop a more severe disease in two-year-old animals when infected with the Ball 3 strain of *E. ruminantium* [23].

Pathogenesis with one or more of the sub-Saharan African strains of *E. ruminantium* may be different in goats and similarly in cattle and white-tailed deer. Additional investigations are important in determining the impact of *E. ruminantium* tick transmission by *A. variegatum* and by other vectors likely serving as competent vectors in nonendemic regions, such as *A. maculatum* [21]. Tick transmission is known to alter the infection course for rickettsial infections compared to needle inoculation methods [24,25]. For example, we reported earlier that in a related *Ehrlichia* (*E. chaffeensis*), the pathogen infection challenge to a host from ticks or tick cell culture-grown bacteria induce different host responses compared to vertebrate cell cultured organisms [25,26].

It is yet to be determined how pathogenic are *E. ruminantium* strains circulating in the three Caribbean islands (Guadeloupe, Marie-Galante, and Antigua), where the pathogen is well established. Despite the pathogen presence in the three neighboring islands for two centuries, heartwater is not known to cause any major outbreaks in ruminant populations on the islands. Also, despite the widespread distribution of *A. variegatum* in several Caribbean islands [11,15], *E. ruminantium* distribution is restricted only to the three islands where it was originally found. It is, therefore, logical to hypothesize that *E. ruminantium* strains in the Caribbean islands are less virulent. Likewise, *A. variegatum* ticks found in several Caribbean islands are likely well-adapted in resisting *E. ruminantium* infections. It will be of considerable interest testing these two hypotheses to gain clear understanding of the disease risk to ruminant populations in North, Central, and South Americas. 

Heartwater remains a very important tick-borne disease of ruminants due to great economic loss it causes to farmers in many countries within the sub-Saharan African region where the disease is endemic [5,9]. The most effective means of controlling heartwater is by reducing the tick burden on animals with the use of acaricides [27]. Additionally, to reduce the disease risk in parts of Africa, a vaccine is offered where animals are inoculated intravenously with live *E. ruminantium*-infected sheep blood and then subjected to antibiotic treatment during the clinical phase [28,29]. This vaccine has three major limitations; (a) it does not offer good protection against heterologous strains, (b) infected blood may carry other pathogens impacting the ruminant health, and (c) it cannot serve as a vaccine for ruminants in non-endemic regions due to the risk of introducing the disease. While several researchers described good protection when *E. ruminantium* whole cell inactivated antigens are used in the vaccine formulations [2,30,31], progress is yet to be made for field applications. Despite these efforts, much remains to be defined in developing a vaccine that provides the best protection and its application to reduce the heartwater burden and risk in endemic and nonendemic regions, respectively. Considering the lack of effective means of controlling the disease, including the lack of rapid diagnostics, heartwater remains a major economic threat to regions where the disease is nonendemic. 

There are no reports in the literature describing the true impact of heartwater to ruminant population in the USA and similarly in other nonendemic regions, while its introduction is considered as a serious threat by the USDA. The likelihood of introduction of heartwater cannot be ruled out via importation of infected tick vectors through exotic animals brought from Africa or from the Caribbean islands. It is unclear how much damage heartwater may inflict on ruminant populations if pathogenic *E. ruminantium* strains were to be introduced into the USA. Our current study is the first attempt in assessing pathogenesis of this important pathogen in animals reared in a nonendemic region.

We presented the first evidence that infection of *E. ruminantium* strains originating from Africa in sheep reared in North America cause pathogenesis with a high degree of severity although they did not result in fatal disease. If *Amblyomma* species ticks carrying *E. ruminantium* were introduced to the USA through the importation of exotic animals from Africa or Caribbean, then these ticks may well be adapted and spread in parts of the USA where conditions are ideal for the tick establishment [20]. Other possible mean of *A. variegatum* introduction from the Caribbean to the USA mainland is via cattle egret (*Bubulcus ibis*) migrations, as this bird species is known to harbor immature stages of the tick [20,32,33]. Nonetheless, no data are available regarding the spread of *E. ruminantium* through immature stages of ticks carried by cattle egrets. Similarly, it is unclear how pathogenic *E. ruminantium* strains circulating in the Caribbean would be to ruminants in North America. Introduction of exotic turtle ticks positive for *E. ruminantium* is described over two decades ago from Florida [34], although the spread of those ticks is not reported thereafter, possibly due to strict control measures. Our study is important in extending investigations focused on assessing the risk of transmission of *E. ruminantium* from infected ticks imported from the Caribbean and Africa to animals in North America.

Future risks of introduction of *E. ruminantium*-positive ticks originating from parts of Africa and Caribbean cannot be ruled out. Consistent with this hypothesis, recent evidence supports the establishment of the Asian Longhorned tick (*Haemaphysalis longicornis*) in the USA by an undefined origin of introduction [35,36]. There is no clear impact data of this tick to the USA economy; however, its introduction into New Zealand and Australia has resulted in high economic losses to their cattle industries [35,37]. Similarly, if *A. variegatum* or other *Amblyomma* species native to Africa were to be introduced along with *E. ruminantium* into the USA, then heartwater could spread rapidly. If this occurs, nearly 80% of domestic and wild ruminants may be adversely impacted leading to high morbidities and mortalities [13,20]. This would translate to immense economic losses to the USA dairy and beef cattle industry and possibly also to the sheep and goat industries [7]. This is based on the assumption that the *E. ruminantium* infections cause significant morbidity and mortality to USA ruminants. However, there is no data in the literature describing how *E. ruminantium* strains would impact ruminants from USA. Our current experimental infection study is the first in addressing this important query.

Evaluating the heartwater risk to ruminants in the USA requires additional investigations aimed at defining pathogenesis of various *E. ruminantium* strains originating from Africa and the Caribbean where the pathogen is more prevalent. Furthermore, preparedness in preventing the introduction of this high-risk disease into the USA should also involve research in developing vaccines effective in protecting against all major strains of the pathogen. Vaccines efficacious in controlling heartwater are also vital in improving the economy of many sub-Saharan African countries where the disease continues to threaten the livelihood of numerous small farmers, besides aiding in reducing the risk of spreading the pathogen to non-endemic regions. Also desirable is the development of a sensitive and specific laboratory toolkit necessary to monitor introduction of *E. ruminantium* from animals and vectors known to transmit the pathogen into non-endemic regions. The current study represents the first in demonstrating severe heartwater pathogenesis with seven different strains of *E. ruminantium* in sheep reared in North America.

## 4. Materials and Methods

### 4.1. E. ruminantium Infected Blood Stabilates

The blood stabilates used in the study were prepared from clinically ill sheep infected with six different strains *of E. ruminantium*: Gardel, Crystal Springs, Nigeria, Malelane, Pokuase, and Highway. The blood stabilates were prepared with the inclusion of 10% DMSO as the cryopreservant using blood recovered from sheep infected in South Africa. The stabilates were shipped in dry ice and stored in liquid nitrogen at the National Veterinary Services Laboratory (NVSL) at Ames, IA for about 15 years. (The stabilates were prepared by the University of Florida research team and shared with the NVSL.) The stabilates were subsequently transported on dry ice to the Kansas State University BSL3 labs with an USDA import permit and stored at −80 °C for few days prior to using for infection experiments. 

### 4.2. Propagation of E. ruminantium in Bovine Pulmonary Artery Endothelial Cells

Bovine pulmonary artery (BPA) endothelial cells and frozen stocks of *E. ruminantium* Crystal Springs, Highway and Mbizi strains were obtained from Agricultural Research Council, Pretoria, South Africa by following similar transportation protocol as above for blood stabilates. Culture stocks were stored in liquid nitrogen. The BPA endothelial cells were propagated in bovine endothelial cell culture medium (Sigma, St. Louis, MO, USA) as per the manufacturer recommended protocols. The *E. ruminantium* strains were propagated in BPA cells as described earlier [38]. Briefly, prior to infection, the endothelial cell culture medium was replaced with L-15 medium supplemented with 0.45% D-Glucose, 10% tryptose phosphate buffer, 5% FBS, and 2 mM L-glutamine. The flasks were maintained until the culture reached 90% infection and then sub cultured to new flasks. The passage level at the time of animal study for Crystal Springs was 7 and for Highway and Mbizi strains was 8. The bacterial cultures were maintained in BPA cells for approximately 15–23 days in each passage at a BSL-3 laboratory, Kansas State University (KSU).

### 4.3. Quantitation of the Cultured E. ruminantium by Real-Time PCR

Quantitation of bacteria in the culture was assessed using a previously published TaqMan™ assay targeting the GroEL gene of *E. ruminantium* [39]. Quantitative results were based on 10-fold serial dilutions of a plasmid encoding an approximately 400 bp region of the groEL gene. The primers used for the groEL gene fragment-based quantitative PCR were prepared as per Sayler et al., 2015 [39]. The reaction contained 500 nM of primers and 250 nM of FAM-labelled *E. ruminantium* groEL PCR product-specific internal probe. The PCRs were performed using the ABI 7500 Real-Time PCR system and accompanying software (Life Technologies, Grand Island, NY, USA). The thermal cycler program included 95 °C for 10 min followed by 40 cycles of 95 °C for 15 s, 55 °C for 60 s, and 72 °C for 30 s.

### 4.4. Animal Inoculation

All animal experiments were conducted at the BSL-3Ag animal facilities at KSU. Experiments with sheep complied with the Public Health Service (PHS) Policy on the Humane Care and Use of Laboratory Animals, the USDA Animal Welfare Act & Regulations (9CFR Chapter 1, 2.31), and were performed with the approval of the KSU Institutional Animal Care and Use Committee. Twelve cross bred sheep of both sexes (six-month-old) were used for the inoculation experiment. All animals were acclimated at a BSL-1 facility to adapt to the laboratory diet for about 10 days and then moved to BSL-3 indoor facility to acclimate for a week prior to the infections. The infection experiments were carried out as two separate studies. In the first experimental infection, 6 male sheep of the Dorper cross-bred breed were inoculated intravenously with *E. ruminantium*-infected blood stabilates of Gardel, Crystal Springs, Nigeria, Malelane, Pokuase, or Highway strains. To take into account of the effect of sex and breed variations on the experimental outcome, the second study included female Katahdin-Romanov crossbred sheep. These animals were inoculated with 5 mL of 90% infected culture derived inoculum, which contained about 4–5 × 10^8^ culture-derived bacteria per inoculum. The blood stabilates and culture inocula were verified for the presence of *E. ruminantium* using *pCS20* nested PCR as described in Molia et al., 2008 [40]. Animals in both infection groups were inspected twice daily for behavioral changes and body temperature changes starting form the second day of infection and the assessment continued for four weeks. Rectal temperature 40.5 °C or higher were considered as fever. At the end of the study period of four weeks, all animals were euthanized in accordance with the recommendations of the Panel on Euthanasia of the American Veterinary Medical Association (AVMA). A commercial euthanasia solution, Fatal-Plus^®^, of volume 0.22 mL/kg containing 86 mg/kg of pentobarbital was administered intravenously.

### 4.5. Necropsy, Tissue Sample Collection, and Histopathology Analysis

On day 28, all animals were euthanized and necropsies were performed by board certified pathologists (coauthors; AGC and CKG or JH). Brain, lung, liver, spleen, kidney, heart, aorta, mediastinal or aortic lymph nodes and other tissues were collected and assessed for gross lesions; and then, subjected to histopathology and molecular analysis. The tissue samples were fixed in 10% formalin for 24 h and processed in a Tissue-Tek^®^ VIP^®^ 6 Vacuum Infiltration Processor (Sakura Finetek, CA). The processed tissues were embedded in paraffin and 5 μm thickness hematoxylin and eosin (H&E) sections were prepared based on standard protocols routinely followed at the Kansas State Veterinary Diagnostic Laboratory (KSVDL). All histopathological observations were evaluated by the board-certified anatomic pathologists (coauthors). Lesions were assessed on a numerical scale of 0 to 3 for each tissue based on the severity and distribution of the lesions in two sections of each tissue measuring approximately 2 cm^2^. The numerical grading scale used was as follows: (1) mild (few cellular infiltrates), (2) moderate (intermediate), and (3) prominent/severe (extensive cellular infiltrates).

### 4.6. Evaluation of Sheep Blood and Tissue Samples for E. ruminantium Detection by PCR and Culture Recovery Methods

At necropsy, brain, lung, liver, spleen, heart, aorta, mesenteric or aortic lymph nodes and other tissues with gross lesions were also collected and processed for PCR assays. Tissue samples for PCR analysis were transferred to −20 °C immediately until use. About 2 mL each of blood collected in EDTA tubes twice a week and on days animal exhibited fever and clinical signs also were used for DNA isolation. Total DNA from approximately 20 mg each of tissue sample or 200 μL of whole blood was isolated using Qia Amp DNA mini kit (Qiagen^®^) as per manufacturer’s instructions. The recovered DNA was resuspended in 200 μL of elution buffer and 2 μL of the final purified DNA was then used for the first round PCR. PCRs were performed using primers targeting the *pCS20* gene of *E. ruminantium* described in Molia et al., 2008 [40]. Briefly, the first round PCR was carried out in a 25 μL reaction volume using Go Taq^®^ Green master mix (Promega, Madison, WI, USA). The PCRs were performed in a Bio-Rad T100™ Thermal Cycler (Biorad, Hercules, CA, USA). The nested PCRs were performed using 2 μL of 1:100 diluted products from the first PCR and using the nested PCR primer sets. For the first round PCR, annealing temperature used was 50 °C. For the second round PCR, the primers were annealed at 55 °C. Stringent protocols and controls were included in all procedures to prevent PCR product contaminations. The culture recovery experiments were also performed as described previously for other *Ehrlichia* species [41]. Briefly, buffy coats were recovered from about 2 mL of whole blood after removing the plasma and lysing RBCs and used to inoculate bovine pulmonary artery endothelial cell cultures. The cultures were monitored for 4–6 weeks for the replicating *Ehrlichia* in phagosomes.

### 4.7. Enzyme Linked Immunosorbant Assay (ELISA)

ELISA assays were performed to determine the development of *E. ruminantium*-specific total IgG in plasma samples collected at weekly intervals till the end point of the study. *Ehrlichia ruminantium* antigens were prepared from Crystal Springs strain cultures. ELISA assays were performed by following the protocol we described earlier for *Ehrlichia chaffeensis* [26,41]. Briefly, the 96-well Immulon 2HB ELISA plates (Thermo Fisher Scientific, Waltham, MA, USA) were coated with purified *E. ruminantium* antigens at a concentration of 20 ng/well using 50 mM sodium carbonate buffer, pH 9.6. One-hundred microliters of each plasma sample starting from day zero (prior to infection) and different days post-infection were diluted 1:50 in phosphate buffered saline containing 0.05% Tween 20 (PBST). The diluted plasma samples were added to the antigen-coated wells and incubated for 2 h at room temperature. The wells were washed three times with PBST and incubated with horseradish peroxidase-conjugated donkey anti-sheep total IgG (Novex by Life Technologies, Frederick, MD, USA) at a dilution of 1:10,000. Unbound secondary antibodies were removed by washing with PBST, and the specific interactions were assessed by color development using TMB (3,3′,5,5′-tetramethyl benzidine) (Calbiochem, San Diego, CA, USA) as the substrate. The ELISAs were performed in triplicate wells and the average absorbance values were used for the analysis.

### 4.8. Statistical Analysis

Statistical analysis was carried out to assess differences in IgG response in each animal before and after infection with *E. ruminantium* strains using a 2-tailed unpaired Student’s t test (GraphPad Software, La Jolla, CA, USA).

## 5. Conclusions

Evaluating the risk of heartwater to animals in non-endemic regions, such as the USA, remained a major challenge due to the limited options of conducting studies on this important disease. The current study represents the critical first step in extending investigations to define heartwater pathogenesis in diverse ruminant populations as well as in developing methods of prevention and control. Additional investigations can now be performed in defining pathogenesis of various strains of *E. ruminantium* originating from the two important geographic regions (Africa and Caribbean) where the pathogen strains are more prevalent.

## Figures and Tables

**Table 1:** Clinical observations in sheep infected with blood with six different blood stabilates of *E. ruminantium* strains.

Sheep Numbers	Strain	Highest Body Temperature Observed	First Day of Clinical Signs	Clinical Signs	Duration of Clinical Signs in Days
**45**	Gardel	39.4 °C	15	Labored breathing, depression	5
**46**	Crystal Springs	39.6 °C	15	Labored breathing, depression	6
**47**	Nigeria	39.7 °C	15	Labored breathing, depression, coughing, mucopurulent discharge	13
**48**	Malelane	39.7 °C	15	Labored breathing, depression	3
**49**	Pokuase	40 °C (2 days)	14	Labored breathing, depression, coughing	8
**50**	Highway	39.6 °C	15	Labored breathing	2

**Table 2:** Gross lesion observations documented during necropsy in sheep infected with six different blood stabilates of *E. ruminantium* strains.

Sheep Numbers	Strain	Larynx	Trachea	Lungs	Heart/Pericardium	Other
**45**	Gardel	Edema mucus	mucus in trachea and bronchi			
**46**	Crystal Springs			Diffuse pulmonary edema		Liver-edema Edema of Tracheobronchial lymph nodes
**47**	Nigeria			Diffuse pulmonary edema	Moderate hydropericardium	Mucus in the oral cavity
**48**	Malelane	Petechial hemorrhage	Hemorrhage	Diffuse pulmonary edema		
**49**	Pokuase		Copious amount of frothy mucus	Diffuse pulmonary edema	Mild hydropericardium	
**50**	Highway			Diffuse pulmonary edema and congestion		Enlarged and edematous mediastinal lymph node

**Table 3:** Clinical outcomes of sheep infected with three different in vitro cultured *E. ruminantium* strains.

Sheep Numbers	Strain	Highest Body Temperature Observed (Duration of Fever in Days)	First Day of Clinical Signs	Clinical Signs	Duration of Clinical Signs in Days
**1**	Crystal Springs	40.3 °C (1)	12	Labored breathing, coughing	4
**2**	Crystal Springs	40.2 °C (1)	9	Labored breathing, depression	3
**3**	Highway	41.1 °C (3)	6	Labored breathing, coughing	7
**4**	Highway	40.2 °C (1)	7	Labored breathing, depression	7
**5**	Mbizi	40.1 °C (1)	12	Labored breathing, depression	4
**6**	Mbizi	40.6 °C (1)	9	Labored breathing, depression, abdominal distension	14

**Table 4:** Gross lesion observations documented during necropsy in sheep infected with in vitro cultured strains of *E. ruminantium*.

Sheep Numbers	Strain	Larynx	Trachea	Lungs	Heart/Pericardium	Other
**1**	Crystal Springs	Lymphoid proliferation in arytenoid	Frothy mucus in trachea and bronchi	Congested, edema and copious amount of frothy mucous in the right lung	Subserosal hemorrhage in the atrium	Mediastinal lymph nodes-enlarged Retro parietal LN-enlarged and congested Spleen- congested
**2**	Crystal Springs		Petechial hemorrhage		Petechial hemorrhage on the pericardial surface of heart	
**3**	Highway			congested		
**4**	Highway		congested		Enlarged and hemorrhagic aortic LN	Enlarged and hemorrhagic mediastinal LN
**5**	Mbizi					Slightly enlarged mediastinal LN
**6**	Mbizi		Frothy mucus and petechial hemorrhage	Filled with white mucous, congested, jelly like consistency	Subserosal petechial hemorrhage around auricles and ventricles	Subserosal hemorrhage in the thoracic wall Liver-congested around margin Hemorrhagic mammary LN and congested mammary tissue Congested conjunctiva MLN, bronchial LN, aortic LN-enlarged and hemorrhagic

**Table 5:** Infection status of tissue samples for sheep infected with *E. ruminantium* determined by nested PCR.

	Sheep	Liver	Heart	Aorta	Mediastinal Lymph Node	CNS	Aortic Lymph Node
Crystal Springs	1	−	+	−	+	−	−
	2	−	−	+	−	+	+
Highway	3	−	−	−	−	+	−
	4	−	+	−	+	−	
Mbizi	5	−	−	−	+	−	
	6	−	−	−	−	−	+

## Data Availability

Not applicable.

## Supplementary Materials

The following are available online at https://www.mdpi.com/article/10.3390/pathogens10040451/s1. Figure S1: White Dorper breed sheep; 6-month-old. Figure S2: *E. ruminantium* in vitro cultured strains. Figure S3: Katahdin X Romanov cross breed; 6 months of age.
