# Supplementary material for: Experimental Infection of North American Sheep with Ehrlichia ruminantium
Source: Pathogens. 2021 Apr 9;10(4):451. doi: 10.3390/pathogens10040451 (PMC8070521; doi:10.3390/pathogens10040451)

Figure S1 (White Dorper breed sheep; 6-month-old)

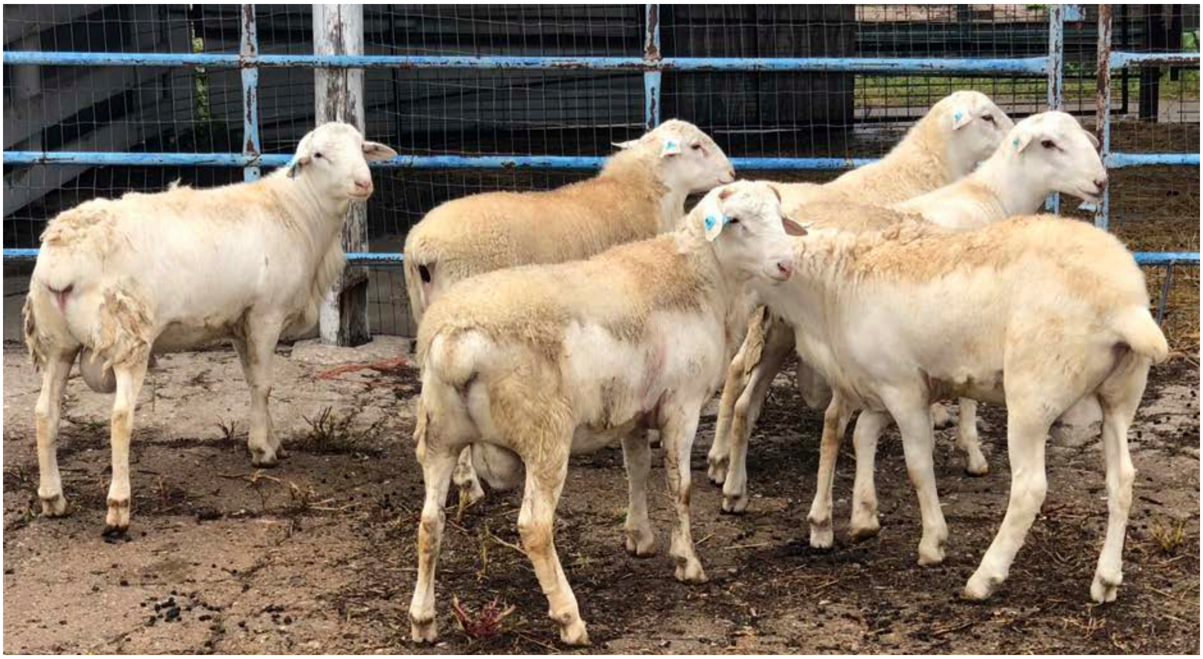

Figure S2 (*E. ruminantium in vitro* cultured strains)

Crystal Springs

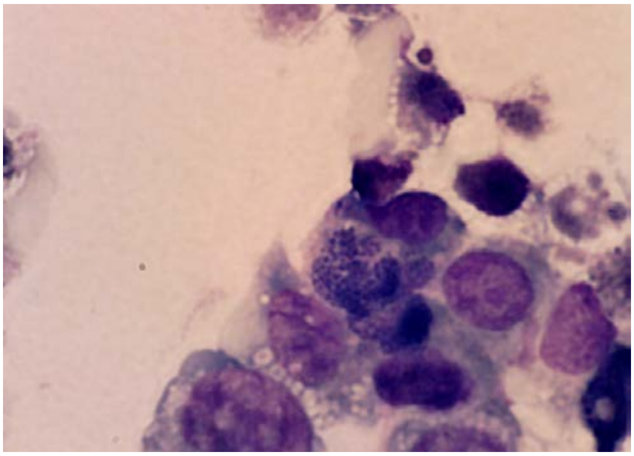

Highway

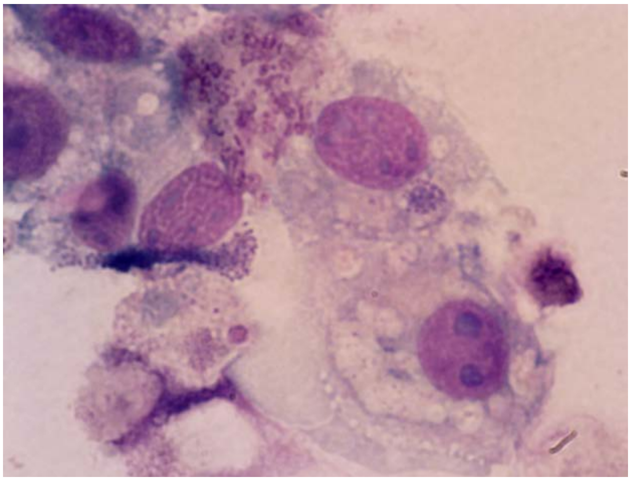

Mbizi

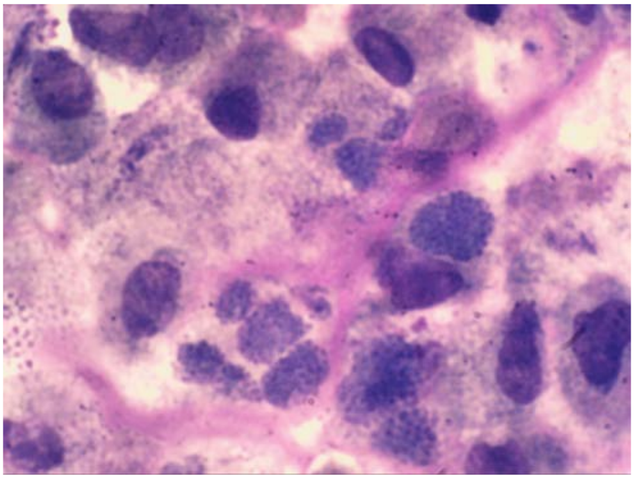

Figure S3 (Katahdin X Romanov cross breed; 6 months of age)

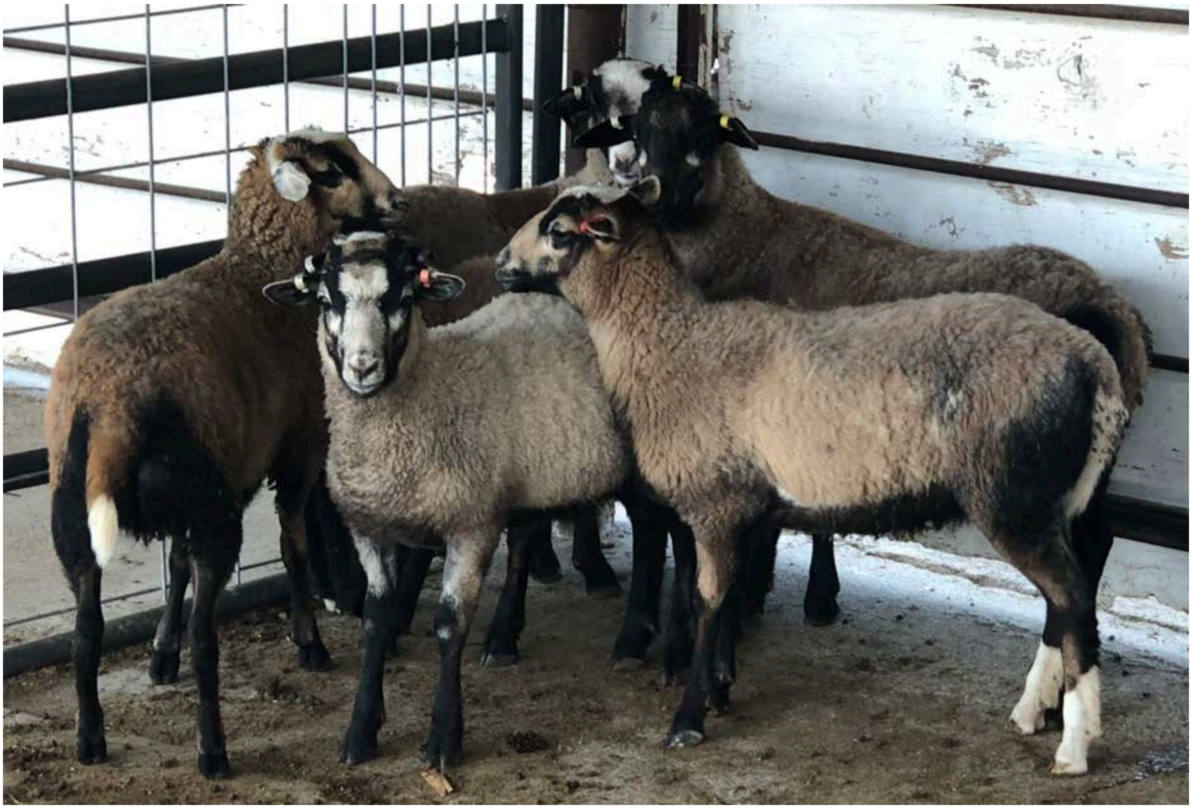

Supplement: Supplementary file 1 [file pathogens-10-00451-s001.pdf]
